# China Expert consensus on the application of metagenomic next-generation sequencing for the etiological diagnosis of infections in hematological disorders (2024)

**DOI:** 10.1097/BS9.0000000000000241

**Published:** 2025-08-29

**Authors:** Chunhui Xu, Ren Lin, Ye Bai, Yanqiu Han, Jianda Hu, Jiong Hu, Yu Hu, Fen Huang, Xiaojun Huang, Chunyan Ji, Xin Li, Aibin Liang, Peihua Lu, Jun Ma, Heng Mei, Ting Niu, Jian Ouyang, Wenbin Qian, Jimin Shi, Yongping Song, Aining Sun, Yehui Tan, Hui Wang, Jianxiang Wang, Yu Wang, Depei Wu, Zhijian Xiao, Ting Yang, Cheng Zhang, Xi Zhang, Xiaohui Zhang, Weili Zhao, Zhuanzhen Zheng, Zunmin Zhu, Sizhou Feng, Qifa Liu

**Affiliations:** a State Key Laboratory of Experimental Hematology, National Clinical Research Center for Blood Diseases, Haihe Laboratory of Cell Ecosystem, Institute of Hematology & Blood Diseases Hospital, Tianjin 300020, China; b Department of Hematology, Nanfang Hospital, Southern Medical University, Clinical Medical Research Center of Hematological Diseases of Guangdong Province, Guangzhou 510515, China; c Tianjin Union Precision Medical Diagnostic Co., Ltd, Tianjin 300020, China; d Affiliated Hospital of Inner Mongolia Medical University, Hohhot 010050, China; e Department of Hematology, the Second Affiliated Hospital, Fujian Medical University, Quanzhou 362000, China; f Department of Hematology, Ruijin Hospital, Shanghai Jiao Tong University School of Medicine, Shanghai 200025, China; g Institute of Hematology, Union Hospital, Tongji Medical College, Huazhong University of Science and Technology, Wuhan 430030, China; h Peking University People’s Hospital, Peking University Institute of Hematology, National Clinical Research Center for Hematologic Disease, Beijing Key Laboratory of Hematopoietic Stem Cell Transplantation, Beijing 100044, China; i Department of Hematology, Qilu Hospital, Shandong University, Jinan 250012, China; j Department of Hematology, the Third Xiangya Hospital of Central South University, Changsha 412000, China; k Department of Hematology, Tongji Hospital of Tongji University, Shanghai 200065, China; l Hebei Yanda Lu Daopei Hospital, Langfang 065201, China; m Institute of Hematology and Oncology, the First Hospital of Harbin, Harbin 150010, China; n Department of Hematology, Institute of Hematology, West China Hospital, Sichuan University, Chengdu 610041, China; o Department of Hematology, Nanjing Drum Tower Hospital, Affiliated Hospital of Medical School, Nanjing University, Nanjing 210008, China; p Department of Hematology, the Second Affiliated Hospital, College of Medicine, Zhejiang University, Hangzhou 310009, China; q The Center of Hematology and Bone Marrow Transplantation, the First Affiliated Hospital, School of Medicine, Zhejiang University, Hangzhou 310003, China; r Department of Hematology, The Affiliated Cancer Hospital of Zhengzhou University, Henan Cancer Hospital, Zhengzhou 450008, China; s The First Affiliated Hospital of Soochow University, Jiangsu Institute of Hematology, Suzhou 215006, China; t Department of Hematology, The First Hospital of Jilin University, Changchun 130031, China; u Department of Clinical Laboratory, Peking University People’s Hospital, Beijing 100044, China; v The Second Department of Hematology, National Regional Medical Center, Binhai Campus of the First Affiliated Hospital, Fujian Medical University, Fuzhou 350209, China; w Medical Center of Hematology, Xinqiao Hospital, Army Medical University, Chongqing 400037, China; x The Second Hospital of Shanxi Medical University, Taiyuan 030001, China; y Department of Hematology, Henan Provincial People’s Hospital, Zhengzhou 450003, China

**Keywords:** Hematological disorders, Metagenomic next, generation sequencing

## Abstract

Infections are frequent complications in patients with hematological disorders, and pathogen diagnosis remains challenging. Metagenomic next-generation sequencing (mNGS) is an unbiased high-throughput technology that has been widely applied in the diagnosis of infectious diseases. However, to date, there are no established international guidelines or expert consensuses regarding the use of mNGS to diagnose infections in patients with hematologic disorders. The Anti-Infection Study Group of the Chinese Society of Hematology invited experts in the fields of hematology, microbiology, and mNGS technology to draft an expert consensus focused on clinical indications, sample collection, quality control, and interpretation of results. This consensus will likely contribute to clarifying the medical indications for mNGS testing, optimizing the interpretation of reports, and becoming an inspiration for global practice.

Patients with hematological disorders often experience immunosuppression because of underlying diseases and/or treatment, and infections are frequent complications.^1–3^ In this population, the spectrum of infectious pathogens is broad, with multidrug-resistance, rare pathogens, and polymicrobial infections being common.^4–7^ This poses a significant challenge for the diagnosis of pathogens. Etiological diagnosis is critical for optimizing anti-infection treatments and improving the prognosis of this population. Unfortunately, conventional microbiological tests (CMTs) are usually time-consuming and have low sensitivity. Metagenomic next-generation sequencing (mNGS) is an unbiased, high-throughput technology that has been widely applied in the diagnosis of infectious diseases.^8–10^ In patients with hematological disorders, mNGS offers several advantages, including high sensitivity, minimal impact of antibiotics, and broad coverage of potential pathogens.^11,12^ However, to date, there are no established international guidelines or expert consensuses regarding the use of mNGS to diagnose infections in patients with hematologic disorders. To clarify the medical indications for mNGS testing and optimize the interpretation of reports in this population, the Anti-Infection Study Group of the Chinese Society of Hematology invited experts from the fields of hematology, microbiology, and mNGS technology to revise and draft an expert consensus based on our published work.^13^

## 1. CLINICAL INDICATIONS FOR ORDERING mNGS TESTING FOR PATHOGEN DIAGNOSIS IN PATIENTS WITH HEMATOLOGICAL DISORDERS

**Figure 1.**
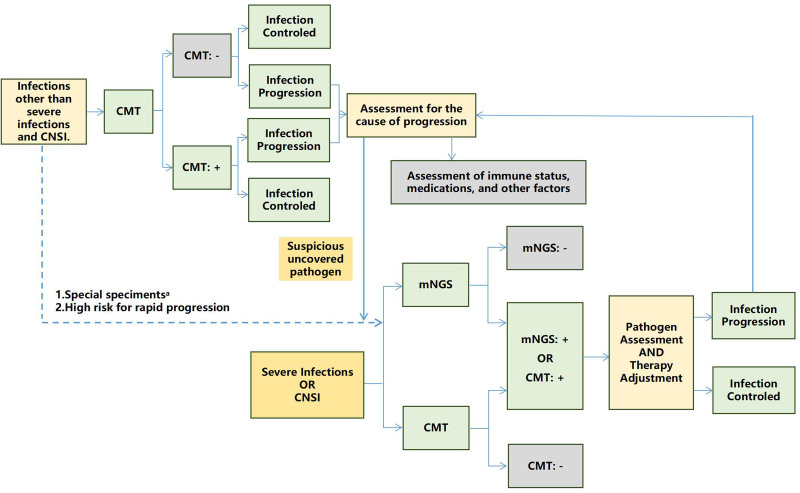
Algorithm for mNGS testing in the patients with hematologic disorders. Special samples include tissues, bone marrow, pus obtained through aspiration, bronchoalveolar lavage fluid, and other sterile samples or samples that are difficult to obtain repeatedly. CMT = conventional microbiological tests, CNSI = central nervous system infections, mNGS = metagenomic next-generation sequencing.

**Consensus 1:** Infections are common complications in patients with hematological disorders. When infectious diseases are suspected, CMTs should be considered the first-line diagnostic approach. mNGS can only be considered in specific circumstances in which CMTs are inconclusive or inadequate (**Fig. 1**).

Infections are common and significant complications in patients with hematological disorders, largely due to the immunocompromised state induced by underlying diseases and treatments, such as chemotherapy and hematopoietic stem cell transplantation. Furthermore, the increasing use of novel therapies, including immunotherapy and molecularly targeted agents, has increased the complexity of the immune landscape.^14–16^ These therapies uniquely affect immune cells and their functions, predisposing patients to different types of infections. When an infectious disease is suspected in hematological patients, it is crucial to select appropriate diagnostic methods based on the patient’s immune status and clinical presentation. The first-line approach for diagnosing infections should remain CMTs, which include culture, microscopy, antigen detection, serology, polymerase chain reaction (PCR) testing, and non-specific infection biomarkers such as C-reactive protein and procalcitonin. However, in cases where CMTs are inconclusive or inadequate, mNGS can be considered a complementary tool. Special attention should be paid to severe infections, central nervous system infections (CNSI), or instances where sample types are difficult to obtain (eg, tissue, bronchoalveolar lavage fluid [BALF], or aspirates). In such scenarios, particularly when empirical therapy fails or infections progress rapidly, a combined approach using CMTs and mNGS is recommended to ensure timely and accurate pathogen identification.

### 1.1. Febrile patients with neutropenia

**Consensus 2:** For low-risk patients without an apparent focus of infections (Table S1, https://links.lww.com/BS/A122), if empirical antimicrobial therapy for ≥7 days shows no significant improvement, it is advisable to collect peripheral blood for mNGS testing in addition to blood cultures. For high-risk patients, if the initial empirical treatment is ineffective within 72 to 96 hours, it is recommended to perform mNGS in addition to CMTs.

Multiple studies have shown that in febrile patients with neutropenia, the diagnostic sensitivity of mNGS was higher than CMTs, especially for those who have received empirical antibiotic treatment.^11,12,17–19^ The use of antimicrobial drugs leads to significantly lower sensitivity of blood cultures, whereas its impact on mNGS is less profound.^11^ Blood can provide a means of pathogen detection.^20^ This method can be beneficial in cases in which local infections result in nucleic acids entering the bloodstream. Patients with neutropenia are prone to invasive fungal infections. When blood mNGS detects organisms such as *Aspergillus* spp. or Mucorales, it often indicates these as causative pathogens, although blood cultures rarely detect them. Blood mNGS provides useful diagnostic information when samples from an infection site are unavailable.^12,21–23^ At the same time, mNGS has advantages in identifying viruses, fungi, and polymicrobial infections.^12,24,25^

### 1.2. Bloodstream infections

**Consensus 3:** For patients with suspected bloodstream infection (BSI), a sufficient volume of peripheral blood should be collected for both mNGS and blood culture. The blood samples should be centrifuged to obtain plasma, which can be temporarily stored at −80°C. If blood culture results are negative within 72 hours and no improvement in the symptoms and signs of infection is observed, plasma mNGS is recommended. For patients with suspected sepsis, it is advisable to submit blood samples for simultaneous mNGS and blood culture.

Multiple studies have shown that combining mNGS with blood culture significantly increases pathogen detection rates.^11,26–28^ Antibiotics can markedly reduce the sensitivity of blood cultures, while their impact on mNGS is much smaller.^11,29^ Additionally, studies have indicated that in confirmed BSIs, mNGS can detect pathogens earlier than blood culture.^12^ Persistent detection of pathogens through mNGS is also associated with the severity and poor control of BSIs.^12,30,31^ Coagulase-negative *Staphylococci* and *Corynebacterium* spp. are common pathogens in catheter-related BSIs.^32,33^ However, they are also skin colonizers, making it difficult to distinguish between colonization and infection using mNGS. Therefore, mNGS is not recommended as a first-line investigation for patients with suspected catheter-related BSI.

### 1.3. Lower respiratory tract infections

**Consensus 4:** For patients with lower respiratory tract infections (LRTI), BALF is the preferred specimen for CMTs. Sputum samples should be considered for patients who cannot tolerate bronchoscopy. Respiratory samples can be stored at −80°C. If anti-infection treatment for ≥72 hours does not lead to improvement of infection symptoms and CMT results are negative, it is recommended to perform mNGS on stored samples. For patients with severe LRTI or those at a risk of rapid progression to severe illness, mNGS and CMTs should be conducted simultaneously.

Some studies have indicated that in LRTIs, the bacterial pathogens are predominantly nonfermentative Gram-negative bacteria.^34^ Additionally, certain difficult-to-culture bacteria such as *Mycobacterium* and *Legionella* are common pathogens in patients with hematological diseases.^35–37^ Fungal pathogens mainly include *Aspergillus* spp., Mucorales, and *Pneumocystis jirovecii*.^22,38–41^ Viral pathogens primarily consist of respiratory viruses, cytomegalovirus (CMV), and adenovirus.^42,43^ Polymicrobial infections are common.^44,45^ mNGS can significantly enhance the pathogen detection rates.^45–49^ Given the broad spectrum of pathogens detected in patients with hematologic disorders and the frequent occurrence of polymicrobial infections, it is recommended to perform mNGS on BALF in addition to CMTs for patients who do not respond to empirical treatment and those with severe LRTI.

Studies have indicated that in proven or probable pulmonary invasive mold infections, the sensitivity of blood mNGS can exceed 50%.^48,50,51^ Additionally, blood mNGS aids in the early diagnosis of invasive pulmonary fungal infections.^52^ In cases of Mucorales infection, the peripheral blood pathogen nucleic acid load is higher than that of *Aspergillus* nucleic acids, making blood mNGS more sensitive for detecting invasive Mucorales infections than for detecting invasive *Aspergillus* infections.^48^ However, for bacterial infections, the sensitivity of mNGS testing using blood samples is significantly lower than that using BALF samples.^53,54^ Although viruses can often be detected in blood samples, especially Epstein-Barr virus (EBV) and CMV, EBV is not usually considered a primary pathogen in pulmonary infections. The diagnosis of CMV-associated LRTI necessitates CMV testing of BALF samples. BALF samples show higher sensitivity than blood samples for nucleic acid detection of SARS-CoV-2.^55^ In addition, sputum can be used to diagnose LRTIs if BALF samples cannot be obtained for testing. In addition, sputum samples are more affected by oral-colonizing bacteria than BALF samples. Additionally, upper respiratory tract specimens cannot reliably predict LRTIs.^56,57^ The accuracy of sputum mNGS testing is lower than that of BALF samples.^58^ Therefore, when selecting sputum for mNGS, it is important to control the quality of sputum samples and interpret the results with caution.

### 1.4. Central nervous system infection

**Consensus 5:** For patients suspected of having CNSI, it is recommended to submit cerebrospinal fluid (CSF) samples for both CMTs and mNGS. DNA testing is advised, whereas RNA testing can only be conducted when an infection with RNA viruses is considered.

CNSI is a common infection in patients with hematological diseases, particularly following allogeneic hematopoietic stem cell transplantation.^59–61^ Multicenter studies have indicated that infections account for nearly one-third of post-transplant neurological complications.^60^ In the early post-transplant period, herpes virus infections are the most common, whereas in the later post-transplant period, bacterial and fungal infections are common.^60,62^ Several studies have demonstrated that mNGS testing of CSF samples can improve pathogen detection rates in CNSI.^63,64^ The advantage of mNGS testing of CSF is its ability to detect multiple pathogens simultaneously, even with a small sample volume. In addition, mNGS can be used to identify polymicrobial infections. Human herpes virus 6, HSV-1, CMV, EBV, and adenoviruses are common types of CNSI. Additionally, rare pathogens such as coronaviruses, astroviruses, hantaviruses, polyomaviruses, and *Toxoplasma* have also been reported in various mNGS case studies.^63,65–68^ In terms of diagnostic performance, mNGS may miss low-load pathogens compared with PCR testing. Some studies have optimized the detection using probe enrichment.^67^ When these tests are not available, it is advisable to submit CSF for both traditional microbiological and mNGS testing.

A negative result from CMTs should not be used as a standard to rule out CNS viral infections.^63,69,70^ The detection of microorganisms by mNGS should be considered carefully, as normal CSF is sterile. Notably, the number of microbial reads does not represent the pathogen load because mNGS is a qualitative test. Viruses like EBV and CMV can exhibit latent infections and thus detection by mNGS does not necessarily indicate an active infection and must be interpreted based on clinical findings. Furthermore, it is common for pathogens to be detectable in the CSF but absent in the peripheral blood. Therefore, for patients with suspected CNSI, it is not recommended to submit peripheral blood samples for mNGS.

### 1.5. Gastrointestinal or intra-abdominal infections

**Consensus 6:** CMTs are recommended as first-line tests in patients with suspected gastrointestinal infections. Stool samples can be submitted for mNGS as an alternative. For patients with suspected intra-abdominal infections, samples obtained from the infection site should be prioritized. Otherwise, peripheral blood may be considered, but with limited value.

Although there have been successful studies using stool mNGS to detect intestinal infections, no studies have specifically targeted patients with hematological diseases.^71,72^ The presence of the gut microbiota poses challenges in interpreting mNGS reports. The detection of obligate bacteria^73^ that are known to cause gastrointestinal infections, such as *Salmonella* spp., *Clostridioides difficile*, *Shigella* spp*.,* and *Vibrio cholerae*, yields clear clinical significance.^73,74^ However, detection of common gut colonizers has limited clinical relevance. Detection of viruses, such as gastroenteritis viruses (rotavirus, enteric adenovirus, and astrovirus), often indicates pathogenicity.^73^ Abdominal candidiasis is a common infection in patients with hematological diseases, but the detection rate of blood cultures is low.^75,76^ Blood mNGS has higher sensitivity than blood cultures and may be used as an alternative sample choice for suspected disseminated candidiasis involving intra-abdominal infection.^77^

### 1.6. Skin and soft tissue infection

**Consensus 7:** For patients with deep or disseminated skin and soft tissue infections (SSTIs), it is recommended to conduct simultaneous mNGS testing in addition to CMTs using specimens obtained from the infection site. Peripheral blood may be an alternative sample type for disseminated SSTIs.

The skin and soft tissues are common sites of infection in immunocompromised hosts. These infections are prone to dissemination or may manifest as disseminated infections. The bacteria that cause these infections include *Staphylococcus aureus*, *Pseudomonas aeruginosa*, *Stenotrophomonas maltophilia*, *Aeromonas* spp., *Nocardia* spp., and nontuberculous mycobacteria. Viruses, particularly human herpes viruses, are common, with the reactivation of latent infections being the most common cause of viral SSTI, mainly herpes simplex virus-1 (HSV-1) and varicella-zoster virus. Fungal pathogens included *Fusarium* spp., *Candida* spp., *Cryptococcus* spp., Mucorales, and *Trichosporon* spp.^78–81^

The advantages of mNGS for diagnosing SSTI include a high positivity rate, broad coverage, and detection of mixed infections.^82^ Samples from the infection site should be prioritized as the detection rate is higher than that of blood. When local samples cannot be obtained from disseminated infections, peripheral blood can be used as an alternative. Samples from both the infection site and blood may be submitted depending on the specific clinical circumstances.

### 1.7. Urinary tract infections

**Consensus 8:** For patients with complicated urinary tract infections (UTIs) or post-transplant urinary system infections, urine mNGS may be considered when CMTs fail to provide etiological evidence or when treatment is ineffective.

Common pathogens include Enterobacteriaceae, *Enterococcus* spp., yeasts, and viruses.^83^ Currently, there are no studies on the application of mNGS for UTI in patients with hematologic diseases. mNGS has advantages in detecting culture-negative infections, mixed pathogen infections, and antibiotic resistance in UTIs.^84–86^ For patients with UTIs who are negative for traditional microbiological tests, urine mNGS may be attempted. If the infection site is the kidneys, biopsy tissue is the first choice for testing. If tissue samples cannot be obtained, blood can be used as an alternative, although the positivity rate is lower than that of tissue samples.

## 2. SAMPLE COLLECTION AND QUALITY CONTROL

**Consensus 9:** For mNGS, samples should be collected directly from the site of infection. Peripheral blood samples may be considered an alternative.

It is recommended that puncture or surgical samples be prioritized based on the site of the infection. If such samples cannot be obtained, efforts should be made to minimize contamination by normal flora and colonizing bacteria when sampling from other sites. Samples should be transported to the laboratory immediately after collection to reduce nucleic acid degradation.^87^ All specimens for mNGS should be selected, collected, and transported to optimize the analysis (Table 1). RNA viruses in samples are prone to degradation; therefore, samples should be tested immediately after collection. If temporary storage is necessary, samples should be stored at low temperatures to preserve RNA integrity. Blood and swab samples can be stored in tubes containing nucleic acid-preserving agents. The pathogen load in the blood is generally low for various types of infections; hence, blood is recommended as an alternative. Blood mNGS may be conducted when no infection focus is apparent, clinicians have difficulty obtaining samples from the infection site, and clinical and laboratory evaluations indicate the presence of pathogens and/or nucleic acids in the blood. The results of mNGS testing depend on the quality of the specimens. Specimen collection should strictly follow aseptic techniques and should be promptly transported to the laboratory for testing. Proper temperatures and containers should be selected for temporary storage and transport to minimize nucleic acid degradation.^87^ Fluid samples are generally collected from the second tube to reduce the interference from skin colonizers at the puncture site.

**Table 1:** Specimen Collection for mNGS Testing.

Specimens	Sample volume	Container	Notation
Plasma	5 mL	Cell-free DNA storage tube	1. When collecting blood, avoid drawing from the same side as an intravenous infusion or simultaneous with a lipid emulsion; immediately after collection, gently invert the sample 8–10 times to mix and reduce hemolysis. 2. Before collection, disinfect the venipuncture site with an appropriate skin disinfectant product, minimize the impact of skin colonizers on the mNGS testing.
Paraffin-embedded tissue sections	10–15 sections	-	Sequencing results from paraffin-embedded tissues are often heavily contaminated; it is recommended to perform testing only when an appropriate negative control standard is selected, and to interpret the results with caution.
Cerebrospinal fuid	2 mL	Sterile container	To reduce contamination by colonizing bacteria, it is recommended to collect the second tube of cerebrospinal fluid.
Aqueous aspirate	≥0.2 mL	Sterile container	-
Aspirate fluid	2 mL	Sterile container	It is recommended to discard the first 2–3 drops and then collect the sample.
Pus	3 mL	Sterile container	-
Tissue	≥3 × 3 × 3 mm^3^	Sterile container	It is recommended to collect samples directly from the site of infection. For skin and soft tissue infections, prioritize tissue from the base of the infection, followed by aspirate (fluid). Freshly collected tissue should not be added to formalin. If the biopsy tissue is too small and prone to drying, a small amount of protective solution or sterile saline can be added to moisten the tissue.
Pleural effusion and ascitic fluid	5–10 mL	Sterile container	To reduce contamination by colonizing bacteria, it is recommended to collect and submit the second tube for testing. Fluid from drainage bags is not recommended for submission.
Urine	5–10 mL	Sterile container	After cleaning the external genitalia, collect a clean-catch midstream urine sample; it is recommended to submit the first morning urine for testing.
BALF	5–10 mL	Sterile container	To reduce contamination by colonizing bacteria, it is recommended to collect and submit the second tube for testing.
Feces	3–5 mL	Sterile container	-
Sputum	3–5 mL	Sterile container	Rinse the mouth 2–3 times, then forcefully cough up deep sputum.
Swab	2–4 swabs	Sterile container	Swab collection should be performed from the base of the infection as much as possible after cleaning the wound.

BALF = bronchoalveolar lavage fluid, mNGS = metagenomic next-generation sequencing.

All specimens should be tested immediately after collection. If temporary storage is necessary, blood can be stored at room temperature for 1–2 d; for longer durations, plasma should be separated and stored. Samples should not be stored at −20°C for more than 1 wk but can be stored long term at −80°C. Dry ice is needed for shipping samples. During the transportation, repeated freeze-thaw and intense shaking should be avoided.

## 3. INTERPRETATION OF RESULTS

**Consensus 10:** If the microorganism detected by mNGS is rarely a colonizer and has proven pathogenicity, it should be considered a potential pathogen, even with low reads.

**Figure 2.**
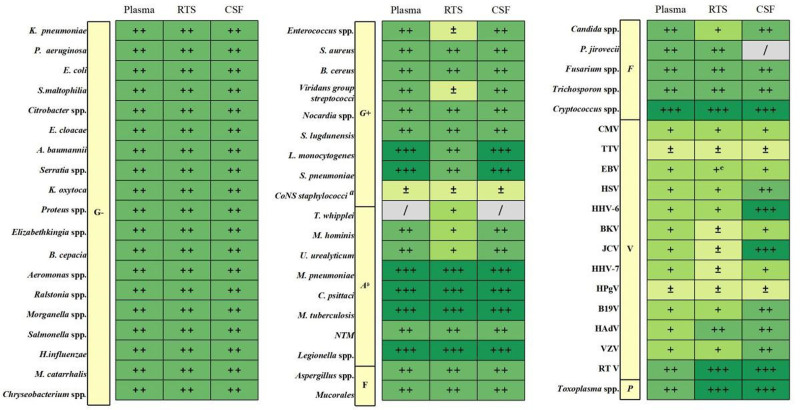
Clinical significance of different microorganisms detected in samples of patients with hematologic disorders by mNGS./ = infection is rare, ± = colonization or no clear pathogenic significance, + = opportunistic pathogen, can be infectious but may also be found in asymptomatic individuals, ++ = opportunistic pathogen, most detections indicate infection, +++ = detection should be considered as pathogenic, negative traditional microbiological testing cannot rule out infection. ^a^Most coagulase-negative staphylococci are colonizers or kitome, with exceptions (like *Staphylococcus lugdunensis*); ^b^Atypical bacteria; ^c^pneumonia caused by EBV is rare, and detection of EBV post-HSCT can be associated with rare PTLD. CSF = cerebrospinal fluid, EBV = Epstein-Barr virus, HSCT = hematopoietic stem cell transplantation, mNGS = metagenomic next-generation sequencing, NTM = nontuberculous Mycobacteria, PTLD = post-transplant lymphoproliferative diseases, RTS = respiratory tract samples, RTV = respiratory tract infection RNA virus.

Microorganisms with known pathogenicity, especially those that can infect immunocompetent individuals, such as *Mycobacterium tuberculosis*, *Cryptococcus* spp., *Legionella pneumophila*, *Chlamydophila psittaci*, and parasites (with *Toxoplasma gondii* being the most common in patients with hematological disorders), should be interpreted as pathogenic.^68,88–90^ These findings should be validated by appropriate methods, including PCR assay, culture, and serological testing.^91^ The clinical implications of the different microorganisms are outlined in Figure 2.

**Consensus 11:** The likelihood of pathogenicity depends on the clinical context in which the microorganisms are detected in aseptic samples. It is important to differentiate between the pathogens and colonizers.

For sterile samples such as blood, tissue, CSF, pleural and abdominal effusions, and aspirates, the detection of bacteria and fungi by mNGS should be interpreted in the context of epidemiology, site of infection, and likelihood of colonization.^92–94^ Empirically, the detection of a single bacterial or fungal species using a large number of sequencing reads suggests a higher likelihood of pathogenicity. However, when multiple microorganisms are detected, colonization needs to be considered in addition to the possible dissemination routes of microorganisms.^92,93,95^ For instance, in gastrointestinal or intra-abdominal infections, the disruption of the intestinal mucosal barrier can lead to the translocation of various gut microorganisms (or nucleic acids) into the bloodstream. Blood mNGS might detect multiple microorganisms, which, on the one hand, should not rule out polymicrobial infections and, on the other hand, might merely represent the entry of nucleic acids into the bloodstream.

**Consensus 12:** Detection of DNA viruses in peripheral blood samples using mNGS is common and does not always indicate latent infection.

In patients with hematological disorders, the detection of viruses using mNGS is common and often involves more than one viral species.^12,51,96^ However, not all detections are indicative of active infection.^97^ Furthermore, viral detection does not indicate reactivation. Therefore, it is necessary to evaluate the viral load using quantitative PCR. If the PCR results are negative, the likelihood of pathogenicity is low, or latent infection should be considered.

**Consensus 13:** mNGS results from non-sterile samples should be interpreted in conjunction with clinical presentation, sample type, and CMT results.

For non-sterile respiratory tract, gastrointestinal tract, and SSTI samples, interpretation (pathogen, colonization, or contamination) should be based on the patient’s clinical presentation, imaging, and other laboratory results.^91^ For instance, *P. jirovecii* can colonize the respiratory tract; therefore, its detection does not necessarily indicate an infection and should be carefully interpreted.^38,98^

**Consensus 14:** The detection of antimicrobial resistance (AMR) genes should be interpreted based on the correlation between genotype and phenotype.

Metagenomic sequencing can be used to identify AMR genes. Correct interpretation depends on the quality of public AMR databases, AMR genetic coverage, and accuracy in determining the bacterial source.^10,99–101^ Some resistance genes may not be expressed; for example, *P. aeruginosa* and *Enterobacter cloacae* chromosomally carry *bla*_AmpC_, which may not be expressed. Therefore, the detection of the *bla*_AmpC_ gene does not indicate resistance.^102^ However, the detection of certain genes in particular bacteria, such as those encoding carbapenemases, is highly indicative of resistance. When Enterobacteriaceaeare detected, *bla*_NDM_ and *bla*_KPC_ genes indicate carbapenem resistance and the *bla*_CTX-M_ gene is related to broad-spectrum cephalosporin resistance; when *S. aureus* is detected, the *mecA* gene suggests β-lactam resistance; when *Acinetobacter baumannii* is detected, the *bla*_OXA-23_ gene indicates carbapenem resistance; and when *Enterococcus faecium* is detected, the *vanA* or *vanB* genes indicates vancomycin resistance.^87,100,101,103,104^

Non-sterile site samples, such as sputum and BALF, often contain multiple microorganisms. Therefore, it is important to consider the bacterial origin when resistance genes are detected. For example, the *mecA* gene could originate not only from *S. aureus* but also from coagulase-negative staphylococci. In addition, the absence of AMR genes does not imply a lack of AMR. This is often due to low coverage of the pathogen, resulting in insufficient sensitivity of resistance genes.

**Consensus 15:** mNGS is a qualitative test and sequence reads do not represent the pathogen load.

For patients with negative culture results whose infection is still not well controlled, mNGS can be conducted to evaluate antibiotic treatment. Persistent detection of a pathogen often indicates that infection has not been fully removed.^12,105^ However, considering that the mNGS methods currently used are only qualitative, the sequence reads do not correspond to the pathogen load. Additionally, because of significant differences in experimental procedures and the proportion of host sequences in different types of samples, comparing the number of reads between samples is not recommended.

**Consensus 16:** A negative mNGS result does not rule out infection.

Although the pathogen detection rate of mNGS is higher than that of CMTs, there is still the possibility of false negatives.^47^ The rationales include the host background in the specimen results in a low proportion of microbial sequences^106–108^; cryptogenic infection foci with a lack of sufficient release of pathogen nucleic acids into the bloodstream (eg, hepatic or splenic candidiasis is characterized by localized infection)^50,108^; the pathogen mainly exists in intact cell form with a lack of free nucleic acids in the blood; thick cell walls of pathogens prevent sufficient lysis for detection, such as with *Cryptococcus* spp. and *M. tuberculosis*^109^; inappropriate or poor quality of the sampling process; and rare infectious pathogens may be missed or misidentified in bioinformatics analysis if they are not included in the database.^9,110^

In summary, mNGS enables the rapid and high-throughput detection of pathogens, providing more comprehensive and unbiased diagnostic clues for patients with hematologic disorders. This is important for the detection of pathogens that are not covered by CMTs or have long turnaround times and low positivity rates when traditional methods are used. In clinical practice, considering the relatively high cost of mNGS, decision-making and sampling should be standardized and limited. It is primarily applied in the diagnosis of acute, severe, critical, and complex infections. CMTs should be the first-line choice and culture remains the gold standard for pathogen detection. mNGS can be a powerful supplement and extension to CMTs, rather than a replacement. Clinicians should fully consider the pathogenicity, epidemiology, and experimental procedures in the interpretation of mNGS reports to make comprehensive decisions based on the clinical characteristics of the patients.

## ACKNOWLEDGMENTS

This work was supported by the Chinese Academy of Medical Sciences Innovation Fund for Medical Sciences (grants 2021-I2M-1-017, 2023-I2M-2-007), the National Natural Sciences Foundation of China (82470208), and the National Key R&D Program of China (2024YFC2510500).

## Supplementary Material


