# Supplementary material for: China Expert consensus on the application of metagenomic next-generation sequencing for the etiological diagnosis of infections in hematological disorders (2024)
Source: Blood Sci. 2025 Aug 29;7(3):e00241. doi: 10.1097/BS9.0000000000000241 (PMC12401339; doi:10.1097/BS9.0000000000000241)
Supplement: Supplementary file 1 [file bs9-7-e00241-s001.pdf]

Supplemental Content

Table S1. Risk Assessment of Febrile Neutropenia Patients

Risk Level	Definition
High Risk	Meeting any of the following criteria:
	1. Expected severe neutropenia (<0.1 x $10^{\wedge 9}$/L) lasting > 7 days.
	2. Presence of any one of the following clinical complications (including but not limited to): □ Hemodynamic instability; □ Oral or gastrointestinal mucositis, causing difficulty in swallowing; □ Gastrointestinal symptoms (abdominal pain, nausea, vomiting, and diarrhea); □ Newly developed neurological or mental alterations; □ Bloodstream or catheter-related infection, especially catheter tunnel infection; □ Newly developed pulmonary infiltrate or hypoxemia, or potential chronic obstructive pulmonary disease.
	3. Liver dysfunction (transaminase levels > 5 times the upper limit of normal) or renal dysfunction (creatinine clearance < 30 ml/min).
	4. Complicated by immunodeficiency diseases.
	5. Receiving immunosuppressive therapy or immune modulating therapy.
Low Risk	Expected neutropenia lasting ≤ 7 days, with no clinical complications, and normal liver and kidney function or only mild and stable impairment.
